# Unraveling the Relationship Between Lipids and Volatile Organic Compounds in *Longissimus Dorsi* of Chuanbai Rex and New Zealand White Rabbits

**DOI:** 10.3390/foods14234024

**Published:** 2025-11-24

**Authors:** Meijun Zeng, Yang Li, Xiulian Wang, Ting Bai, Jie Cheng, Zhoulin Wu, Xiaohua Huang, Bo Wang, Rui Zhang, Jiamin Zhang, Wei Wang

**Affiliations:** Meat Processing Key Laboratory of Sichuan Province, Sichuan Provincial Engineering Research Center of Meat Quality Improvement and Safety Control Technology, College of Food and Biological Engineering, Chengdu University, Chengdu 610106, China; zengmeijun@cdu.edu.cn (M.Z.); m19923603356@163.com (Y.L.); 212023095135068@cdu.edu.cn (X.W.); baiting@cdu.edu.cn (T.B.); chengjie@cdu.edu.cn (J.C.); huangxiaohua@cdu.edu.cn (X.H.); wangbo@cdu.edu.cn (B.W.);

**Keywords:** rabbit breeds, flavor profile, lipid metabolism, multivariate analysis, volatile compounds, meat quality

## Abstract

Chuanbai Rex (CR) and New Zealand white (NZ) rabbits are highly popular and widely produced for meat consumption in Sichuan, China. But comparative studies on nutritional and sensory qualities of meat from two breeds are still lacking. This study integrated lipidomic and volatilomic approaches to elucidate the breed-specific differences in the *longissimus dorsi* between CR and NZ (n = 5 per breed). Analysis of fatty acid composition revealed that CR had lower content of PUFA but with a more balanced n-6/n-3 PUFA ratio compared to NZ. LC-MS-based lipidomics identified 52 significantly different lipids between the two breeds, and CR had higher levels of phospholipids and sphingolipids, while NZ was richer in triglycerides and diglycerides. SPME-GC-MS analysis identified a total of 140 VOCs, including primarily aldehydes (>50%), alcohols (<20%), and hydrocarbons (<15%). CR contained unique aroma compounds such as acetoin and 2-(E)-heptenal, while NZ had more of nonanal and pentadecane. Pearson correlation analysis performed between differential lipid metabolites and characteristic VOCs showed that 22 lipid biomarkers were significantly correlated with seven key VOCs, suggesting breed-distinct pattern for flavor formation. Albeit a small sample size, this study provides preliminary insights into biochemical mechanisms determining rabbit meat quality and offers a scientific basis for developing premium rabbit meat products tailored to consumer preferences.

## 1. Introduction

Rabbit meat is lean and nutritious, characterized by high levels of essential amino acids, minerals (K, P, Mg), and unsaturated fatty acids (25–32 mg/100 g), alongside low fat (~6.8%), cholesterol (59 mg/100 g), and sodium (37 mg/100 g) content [1,2,3,4]. With an energy value similar to common red meats, it is highly recommended for elderly people, adolescents, and pregnant women [5].

Chuanbai Rex (CR) is a local rabbit breed in Sichuan, China [3,6]. It is primarily bred for high-quality fur but also constitutes a notable part of the local meat market [7]. CR demonstrated good production performance, with an average litter size of 7.3, a pre-weaning survival rate of 89.3%, and a body weight of 1786 g at 13 weeks of age [8]. Though the current rabbit meat market is dominated by introduced breeds like Ira, New Zealand white (NZ), and Hyplus, local breeds, including CR, Tianfu Black, and Shuxing#1, have been actively developed under government initiatives through large-scale farming, processing, and cold-chain logistics. However, for now, comparative studies on the nutritional and flavor profiles between local breeds and introduced breeds are very limited. This knowledge gap restricts the development of value-added rabbit meat products and, ultimately, the sustainable development of the local rabbit meat industry.

Fatty acid and lipid profiles critically affect the health value and flavor of rabbit meat, which are directly influenced by the rabbit breed. For example, NZ, a well-established meat rabbit breed, was reported to contain 21–37% monounsaturated fatty acids (MUFA), 21–41% polyunsaturated fatty acids (PUFA), and an n-3/n-6 ratio of around 0.17–0.21 [9,10]. Zhang et al. compared the fatty acid and lipid profiles of *longissimus dorsi* in Fujian Yellow (FJ) rabbits and NZ, and found that FJ had a higher MUFA content than NZ, with characteristic C14:0, C16:1n7, C20:1n9, and C18:3n3 [11]. Króliczewska et al. compared brown hares and domestic rabbits and found that hares had lower SFA and MUFA content and higher PUFA content, contributing to a superior PUFA/SFA ratio, but with an unfavorable n-6/n-3 PUFA ratio [12].

Further, fatty acids and lipids are critical precursors for a wide range of flavor-related volatile organic compounds (VOCs) [3,13,14]; therefore, unraveling the relationship between lipids and flavor formation would provide valuable insights into the molecular mechanisms underlying rabbit meat quality and their flavor characteristics [15,16,17]. Since fatty acids and lipids in rabbit meat are strongly affected by breeds, the corresponding VOCs and meat flavor can vary significantly [18,19,20,21]. In recent years, integrated lipidomics and volatilomics coupled with multivariate analysis have advanced beyond prior univariate approaches by systematically uncovering hidden relationships between lipids and flavors, thereby deciphering the formation mechanisms of lipid-derived flavor in various meat sources [22,23,24,25,26]. Nevertheless, systematic studies aimed at uncovering the molecular basis of lipid degradation–flavor formation among different rabbit breeds are very scarce. For example, Liu et al. investigated the VOCs and lipid metabolites of CR and Hyla under different cooking methods and found 14 key differential VOCs and 184 differential lipid metabolites associated with amino acid and lipid metabolism [27]. Therefore, we hypothesized that CR and NZ would have distinct lipid profiles and contribute to the formation of characteristic VOCs.

This study, for the first time, systematically analyzed the fatty acid profiles, lipid composition, and VOCs in the *longissimus dorsi* muscle from locally bred Chuanbai Rex and imported New Zealand white rabbits, aiming to elucidate the relationship between lipids and VOCs. GC-MS analysis was employed to quantify free fatty acids and VOCs, and UHPLC-ESI-MS/MS-based targeted lipidomics was used for the identification of potential lipid biomarkers. Multivariate data analysis was performed to reveal the breed-specific variations in lipids and VOCs. Further correlation analysis was conducted to elucidate the relationship between lipid composition and flavor characteristics. The novelties of this work lie in the integrated use of LC-MS-based lipidomics and GC-MS-based volatilomics to bridge the knowledge gap between breed-specific lipid profiles and their impact on VOC formation in rabbit meat, which would provide mechanistic insights into flavor formation and support the breed-specific nutritional evaluation and quality improvement of rabbit meat.

## 2. Materials and Methods

### 2.1. Materials

Dichloromethane (≥99.9%, for HPLC, Catalog No.: D809632), methanol (≥99.9%, for HPLC, Catalog No.: M813956), sodium hydroxide (99.9%, Catalog No.: S817991), isopropanol (≥99.9%, for HPLC, Catalog No.: I811945), acetonitrile (≥99.9%, for HPLC, Catalog No.: A800366), formic acid (≥99.9%, for HPLC, Catalog No.: F809862), ammonium acetate (≥99.0%, for LC-MS, Catalog No.: A800966), and 2,4,6-trimethylpyridine (≥99.8%, for GC, Catalog No.: T882069) were purchased from Macklin (Shanghai, China). All chemicals were used without further purification. Ultrapure water (≥18.2 MΩ·cm) was used to prepare solutions.

### 2.2. Animals and Sample Collection

Chuanbai Rex rabbits and New Zealand white rabbits were reared in the Sichuan Academy of Grassland Sciences (Hongyuan county, Aba Tibet and Qiang Autonomous Prefecture). Rabbits were specifically raised for this study and were kept under the same forest-rearing system with the same diet (composition described in our previous work [28]). At 90 days of age, 5 male rabbits with similar body weights (2.5 ± 0.1 kg) were randomly selected from 5 contemporary litters (1 rabbit per litter) and then slaughtered. After removing the skin, head, and feet, the *longissimus dorsi* was collected, immediately frozen in liquid nitrogen, and transported to the lab and stored at −18 °C. A total of 10 rabbit meat samples were collected, which were named as CR for Chuanbai Rex rabbits and NZ for New Zealand white rabbits, respectively. The 10 samples were used as biological replicates for all the subsequent analyses, and one analysis was conducted for each sample.

### 2.3. Analysis of Free Fatty Acids by GC-MS

The extraction of fatty acids in rabbit meat was performed following a previous method with minor revisions [11]. Briefly, 50 mg of rabbit meat was mixed with 1 mL dichloromethane-methanol (1:1, *v*/*v*) and cryogenically ground for 3 min. After sonicating for 15 min, standing at −20 °C for 15 min, and centrifugation at 13,000 rcf for 10 min, 500 μL supernatant was taken and dried under nitrogen gas. Methylation of extracted fatty acids was conducted by incubation with 0.2 mL of sodium methoxide solution (0.5 mol/L) at 60 °C for 0.5 h. The formed methyl esters were extracted by adding 0.2 mL of *n*-hexane, vortexed for 30 s, then centrifuged at 4 °C, 13,000 rcf for 10 min. Then, 100 μL of the supernatant was taken, filtered through a 0.22 μm membrane, and then transferred to a vial for GC-MS analysis.

Analysis of the fatty acid methyl esters (FAMEs) was performed on Agilent 8890 Gas Chromatograph (GC) with 5977B Mass Spectrometer Detector (MSD) system (Agilent Technologies, Santa Clara, CA, USA). Injection volume was 1 μL with split ratio of 100:1. Carrier gas was high-purity nitrogen (0.7 mL/min) with inlet temperature of 230 °C. A CP-Sil 88 capillary GC column (100 m × 0.25 mm × 0.2 μm, Agilent Technologies, Santa Clara, CA, USA) was used with the temperature programmed as follows: 80 °C for 10 min, increase from 80 °C to 180 °C at 10 °C/min, then hold for 6 min, increase from 180 to 200 °C at 1 °C/min, then hold for 20 min, increase from 200 °C to 230 °C at 4 °C/min, and then hold for 10.5 min. The FAMEs were identified and quantitated (expressed as g/100 g of total FAMEs) according to their retention time (CV of retention time < 0.2%) with comparison to the external FAME standards (FAME Mix 37 Component, Agilent Technologies). For quality control (QC), a blank (only solvent) was tested to subtract the background contamination introduced during sample preparation. A pooled QC sample was prepared by combining equal aliquots from all experimental samples and analyzed repeatedly at regular intervals (every 5 samples) to monitor system repeatability.

### 2.4. Targeted Lipidomic Analysis by UHPLC-ESI-MS/MS

Lipidomic analysis of lipids in breed-specific rabbit meat was performed following a previously described method with minor revisions [29]. Briefly, 50 mg rabbit meat sample was mixed with 280 μL methanol-water (2:5, *v*/*v*) and 400 μL methyl tert-butyl ether, then cryogenically ground for 6 min. After sonicating (5 °C, 40 KHz) for 30 min, standing at −20 °C for 30 min, and centrifugation at 4 °C, 13,000× *g* for 15 min, 350 μL supernatant was taken and dried under nitrogen gas. Then, the sample was resuspended in 100 μL isopropanol–acetonitrile (1:1, *v*/*v*) and vortexed for 30 s. After sonicating (5 °C, 40 KHz) for 5 min and then centrifugation at 4 °C, 13,000× *g* for 10 min, the supernatant was taken, filtered through a 0.22 μm membrane, and subjected to lipidomic analysis.

UHPLC-ESI-MS/MS analysis was performed on a Thermo Accucore C30 column (150 × 2.1 mm, 2.6 μm, Thermo Fisher Scientific, Waltham, MA, USA) coupled with an ESI mass spectrometer. The column temperature was set at 40 °C. The mobile phase included the following: (A) acetonitrile–water (1:1, *v*/*v*) with 0.1% formic acid and 10 mM ammonium acetate; (B) acetonitrile–isopropanol–water (10: 88: 2, *v*/*v*/*v*). The flow rate of the mobile phase was set at 0.3 mL/min, which was programmed as follows: linear increase in B from 20% to 85% for 0–20 min, increase in B to 95% for 20–22 min, then hold at 95% of B for 22–35 min, and then equilibrated at 95% of B until 40 min. The injection volume was set at 5 μL. ESI-MS/MS analysis was performed in the positive/negative ionization mode with a capillary temperature of 370 °C. The flow rates of sheath and auxiliary gas were set at 60 and 20 arbitrary units, respectively. Mass spectra were acquired at a resolution of 200–2000 *m*/*z* using normalized collision energies of 20, 40, and 60 eV. Dynamic exclusion was applied to remove some unnecessary information in MS/MS spectra.

### 2.5. Analysis of Volatile Organic Compounds (VOCs) by SPME-GC-MS

Analysis of VOCs in rabbit meat was performed according to previous method [30], using Agilent 7890B GC with 5977A MSD system coupled with solid-phase microextraction (SPME) (Agilent Technologies, Santa Clara, CA, USA). Briefly, 3.0 g minced rabbit meat was mixed with 1 μL internal standard 2,4,6-trimethylpyridine in a 15 mL vial. SPME was performed on a 50/30 μm divinylbenzene/carboxen/polydimethylsiloxane SPME fiber 57298-U (Sigma-Aldrich, San Francisco, CA, USA), following a program of equilibration at 50 °C for 10 min, heating at 75 °C for 45 min, extraction for 20 min, and desorption for 5 min. For quality assurance, the SPME fiber was conditioned according to the manufacturer’s instructions in the GC injector port. Before initial use and between samples, a blank (desorbing the fiber without any sample) was run regularly to confirm the absence of carryover or fiber-derived contaminants. GC separation was performed on the J&W HP-5ms Ultra Inert column (Agilent Technologies, Santa Clara, CA, USA). The carrier gas was helium (99.9999%) with a flow rate of 1.0 mL/min. The injection temperature was 250 °C. The temperature was programmed as follows: equilibrate at 40 °C for 3 min, increase to 70 °C at 10 °C/min, then hold for 1 min, increase to 120 °C at 3 °C/min, then hold for 3 min, and increase to 160 °C at 5 °C/min, then hold for 1 min. The mass spectra were recorded in the electron ionization mode with an electron energy of 70 eV. The ion source temperature was set at 230 °C, the quadrupole temperature at 150 °C, and the detector voltage at 350 V. The mass scanning range was from 40 to 500 *m*/*z*. Samples were analyzed in triplicate to assess the repeatability of the entire SPME-GC-MS analysis.

The identification and qualitative analysis of the volatile flavor compounds were achieved via the NIST Mass Spectral Library (NIST 14). Quantitation of the individual volatile compound was calculated using Equation (1):

(1) $$C_{i}=\frac{\rho \times \nu \times A_{i}}{A\times m}$$

where $C_{i}$ is the content (μg/kg) of the volatile compound, ρ is the mass concentration of the internal standard (2 μg/μL), ν is the volume (1 μL) of the internal standard, $A_{i}$ is the peak area of the volatile compound, *A* is the peak area of the internal standard, and m is the sample mass (0.003 kg).

### 2.6. Analysis of Odor Activity Values (OAVs)

Odor Activity Value (OAV) was used to evaluate the contribution of each volatile compound to the overall flavor profile of the rabbit meat sample, which was calculated according to Equation (2):

(2) $$OAV=\frac{C_{i}}{F_{i}}$$

where $C_{i}$ is the mass concentration (μg/kg) of the volatile compound, and $F_{i}$ is its odor threshold (μg/kg) in water (obtained from http://www.leffingwell.com). Compounds with OAV ≥ 1 are considered key aroma-active constituents, which significantly affect the overall flavor profile.

### 2.7. Data Processing and Multivariate Analysis

Lipidomic data was analyzed using R packages (pheatmap, ropls, ggplot2, v1.6.2) for clustering, correlation, multivariate analysis, and content distribution. Lipidomic data processing was performed using LipidSearch (Thermo Fisher Scientific, Waltham, MA, USA) to generate lipid metabolite profiles. Differential lipid metabolites were identified via t-tests, principal component analysis (PCA), orthogonal partial least squares discriminant analysis (OPLS-DA), and partial least squares discrimination analysis (PLS-DA) with predictive variable importance of projection (VIP) values. KEGG pathway analysis was conducted via the KEGG database (http://www.kegg.jp/) to classify metabolites based on their functional pathways.

Volatile flavor compounds were analyzed using SIMCA 14.1 and TBtools v1.098769 for multivariate analysis of variance, according to our previously reported methods [31]. PCA was performed to analyze differences in VOCs among the rabbit meat from different breeds. The key VOCs with OAV ≥ 1 were further subjected to OPLS-DA and PLS-DA with predictive VIP. Pearson correlation analysis was performed between the differential lipids and volatile organic compounds using Origin 2023.

### 2.8. Statistical Analysis

Data is presented as mean ± standard deviation based on five biological replicates. Statistical analysis of one-way ANOVA and Tukey’s HSD was performed using SPSS 22.0 (SPSS Inc., Chicago, IL, USA), with * *p* < 0.05 considered statistically significant. To control the increased risk of false positives due to multiple hypothesis testing, the Benjamini–Hochberg procedure was used to control the False Discovery Rate (FDR). A significance threshold of FDR < 0.05 was set, and all reported *p*-values were adjusted following this correction. The Benjamini–Hochberg FDR correction ensured the reliability of exploratory findings deemed significant, allowing higher confidence in predictions derived from them and reducing the risk of false discoveries. Due to the small sample size (n = 5) used herein, it should be emphasized that the statistical analyses are used for exploratory data analysis and trend identification rather than for building predictive models.

### 2.9. Ethics Statement

All animal care and experimental procedures were approved by the Animal Care and Use Committee of Chengdu University (Approval No. SSXY600023, Approval Date 20 October 2023) and conducted in full compliance with both institutional guidelines and local legislation. Written informed consent was obtained from all animal owners prior to the inclusion of their animals in the study.

## 3. Results

### 3.1. Medium- and Long-Chain Fatty Acids (MLCFAs) of Chuanbai Rex and New Zealand White Rabbit Meat

**Figure 1:**
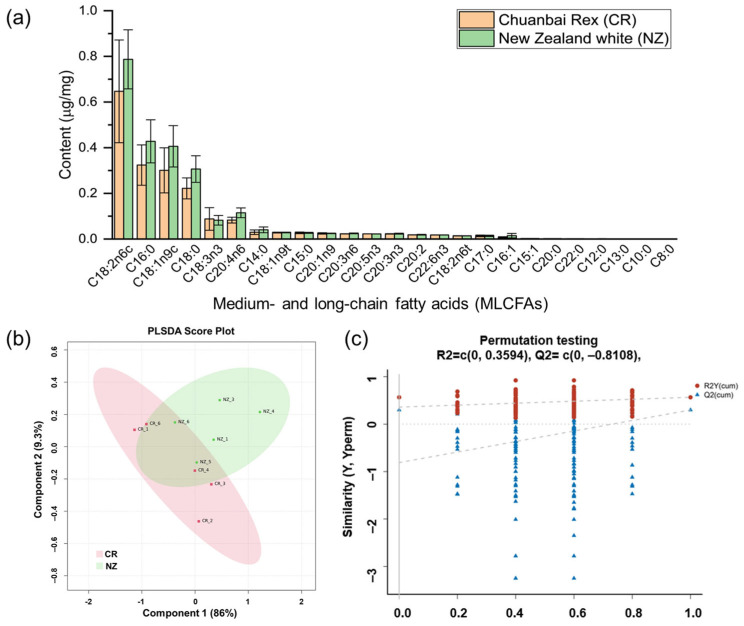
Medium- and long-chain fatty acids (MLCFAs) of *longissimus dorsi* from Chuanbai Rex (CR) and New Zealand white (NZ) rabbits. (**a**) Content (μg/mg) of MLCFAs; (**b**) PLS-DA score plot of CR versus NZ; (**c**) permutation testing plot.

Rabbit meat (*longissimus dorsi*, n = 5 for each breed) from Chuanbai Rex (CR) and New Zealand white (NZ) was analyzed for medium- and long-chain fatty acids (MLCFAs) to compare free fatty acids composition (Figure 1 and Table 1). It was found that the total content of MLCFAs in CR was relatively lower than that in NZ, and the top five abundant MLCFAs were the same for the two breeds, which were linoleic acid methyl ester (C18:2n6c), palmate methyl ester (C16:0), oleate methyl ester (C18:1n9c), stearic acid (C18:0), and α-linoleic acid (C18:3n3). Interestingly, for both breeds, the content of unsaturated fatty acid (UFA) was approximately double that of the saturated fatty acid (SFA), indicating rabbit meat is generally a nutritious source of good fat. Furthermore, compared to CR, NZ was relatively richer in the absolute content of monounsaturated fatty acid (MUFA) and polyunsaturated fatty acid (PUFA). But the n-6/n-3 PUFA ratio in CR was relatively lower than in NZ (5.07 versus 6.44), suggesting CR is an even healthier source of PUFA for human health [32]. From Figure 1b, PLSDA showed a moderate separation between CR and NZ, with a cumulative contribution rate of 95.3% (Component 1, 86%; Component 2, 9.3%). A permutation test with 200 iterations (Figure 1c) validated the model’s robustness (R2Y = 0.3594, Q2 intercept = −0.8108, *p* = 0.01), confirming its stability and reliability without overfitting. In addition, OPLS-DA (Figure S1) showed there were five differential MLCFAs with VIP > 1.0, including C16:0 (VIP = 2.47), C18:1n9c (VIP = 2.30), C18:0 (VIP = 2.29), and C18:2n6c (VIP = 1.59) (Table 1), which can be used to distinguish the fatty acids between the two rabbit breeds.

### 3.2. Comparative Lipidomic Profiling of Breed-Specific Rabbit Meat

#### 3.2.1. Overall Lipid Composition

**Figure 2:**
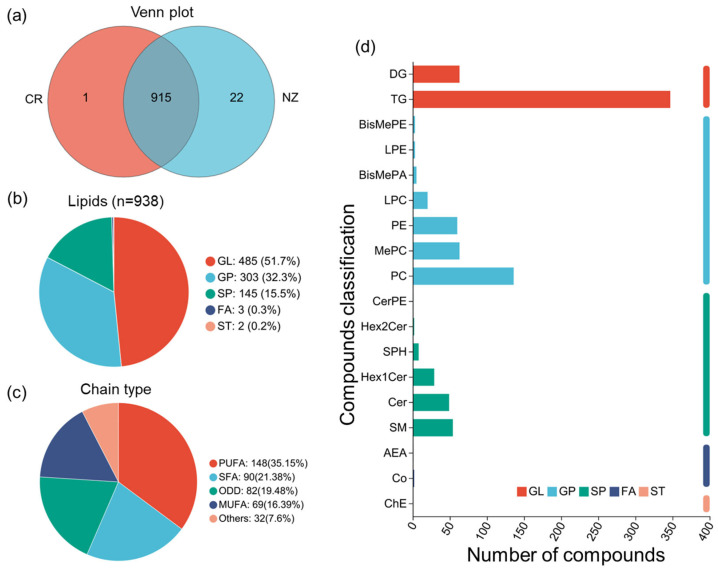
Summary of lipid composition of rabbit meat from Chuanbai Rex (CR) and New Zealand white (NZ). (**a**) Venn diagram; (**b**) lipid category pie chart; (**c**) classification of lipid chain unsaturation; (**d**) subclasses of lipids and the number of compounds.

A total of 938 lipid metabolites were identified in CR and NZ, including 915 lipid metabolites shared between the two breeds, with 1 unique to CR, and 22 unique to NZ (Figure 2a). These metabolites were primarily in the subclasses of glycerolipids (GLs, 51.7%), glycerophospholipids (GPs, 32.3%), and sphingolipids (SPs, 15.5%), with minimal numbers in fatty acyls (FAs, 0.3%) and sterol lipids (STs, 0.2%) (Figure 2b). Analysis of lipid chain unsaturation (Figure 2c) indicated almost balanced percentages of polyunsaturated fatty acyls (PUFAs, 35%) and monounsaturated fatty acyls (MUFAs, 36%). Further analysis of lipids’ carbon numbers (Figure S2) also confirmed the high content of UFAs with more than two double bonds and the presence of long-chain lipids with carbon numbers between 34 and 40. Moreover, lipid classification (Figure 2d) showed that most of the identified lipid metabolites belong to triglycerides (TGs, 347) and phosphatidylcholine (PC, 136), while less-abundant metabolites were classified into methylphosphatidylcholine (MePC, 63), phosphatidylethanolamine (PE, 60), sphingomyelin (SM, 54), and ceramide (CER, 49). Overall, both lipid composition and fatty acids results indicated high percentages of unsaturated fat in both breeds.

#### 3.2.2. Analysis of Differential Lipid Metabolites

**Figure 3:**
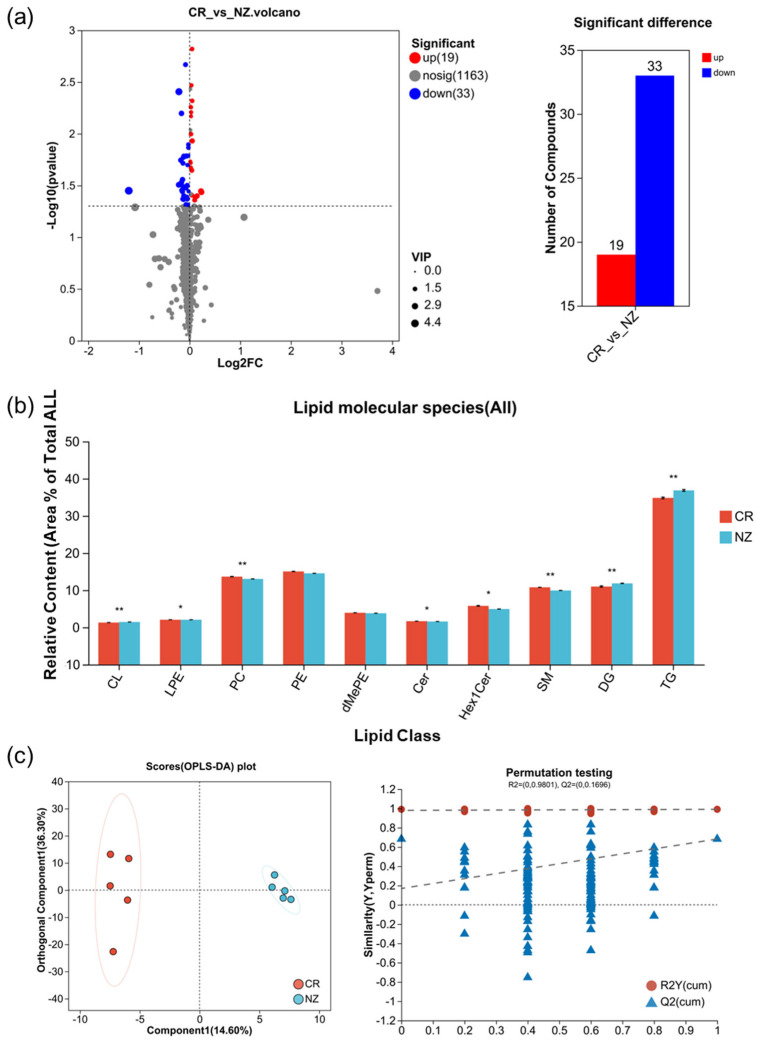
Differential lipid metabolites between rabbit meat from Chuanbai Rex (CR) and New Zealand White (NZ). (**a**) volcano plot with significant difference; (**b**) relative content of lipid class; (**c**) OPLS-DA score plot and permutation testing plot. Statistical differences are denoted by * *p* < 0.05 and ** *p* < 0.01.

As shown in Figure 3a, a total of 52 differential lipid metabolites were identified across CR and NZ, comprising 19 upregulated and 33 downregulated (details in Table S2). These differential lipid metabolites were classified into five subclasses of glycerophospholipids (GPs) (LPE, PE, PC, dMePE, CL), three subclasses of glycerolipids (GLs) (Cer, Hex1Cer, SM), and two subclasses of sphingolipids (SPs) (DG, TG) (Figure 3b). Notably, NZ exhibited significantly higher levels of TG and DG compared to CR, whereas CR had relatively higher levels of PC, Hex1Cer, and SM. To further clarify the metabolite differences between the two breeds, OPLS-DA was applied (Figure 3c). The OPLS-DA score plot revealed a clear separation between CR and NZ, with 14.6% for component 1 and 36.3% for orthogonal component 1. A permutation test with 200 replications yielded R2Y = 0.991, Q2 = 0.978, and R2X = 0.65, indicating the robustness and reliability of the model.

#### 3.2.3. Potential Biomarkers and Metabolic Pathways

**Figure 4:**
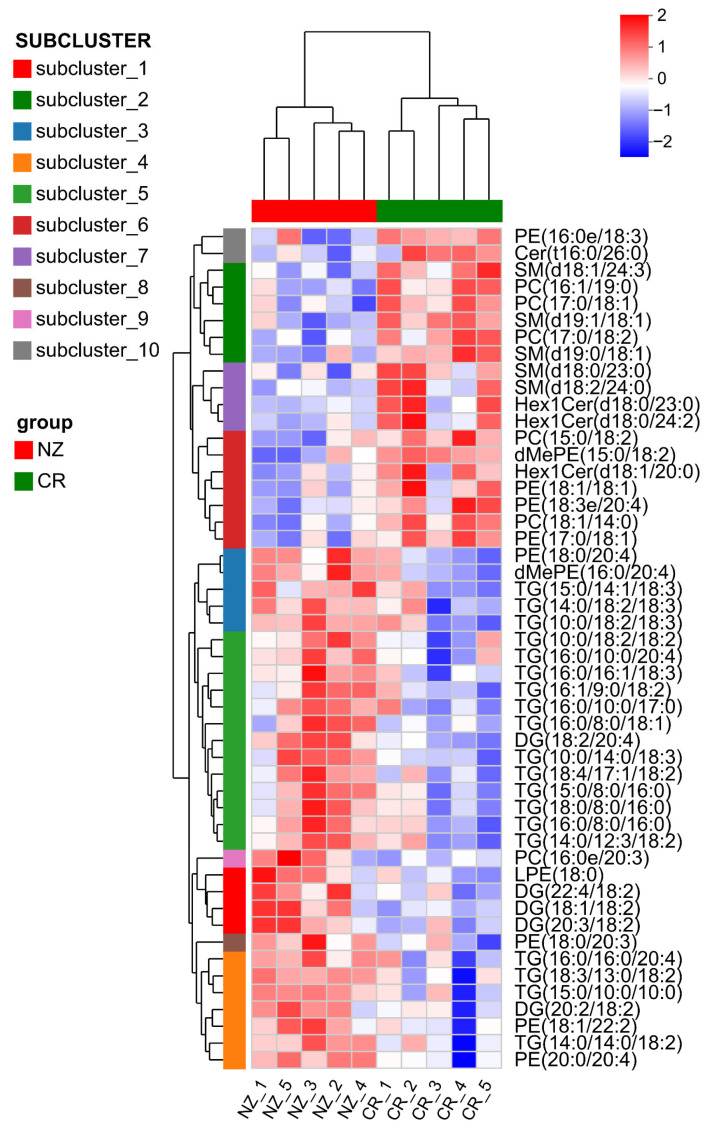
Clustering heatmap of differential lipid metabolites in *longissimus dorsi* from Chuanbai Rex (CR) and New Zealand White (NZ) rabbits for identification of potential biomarkers.

Potential biomarkers of lipid metabolites between CR and NZ were screened via hierarchical cluster analysis. Figure 4 shows distinct clustering of the CR and NZ groups based on the top 50 most abundant lipid metabolites, highlighting significant differences in both metabolite species and abundance. NZ was enriched in TG and DG, while CR had higher levels of phospholipids and SPs, including phosphatidylethanolamine (PE), phosphatidylcholine (PC), sphingomyelin (SM), and glycosylceramide (Hex1Cer). These findings align with the lipid categorization in Figure 3b and validate the clustering analysis for differentiating between the two rabbit breeds.

**Figure 5:**
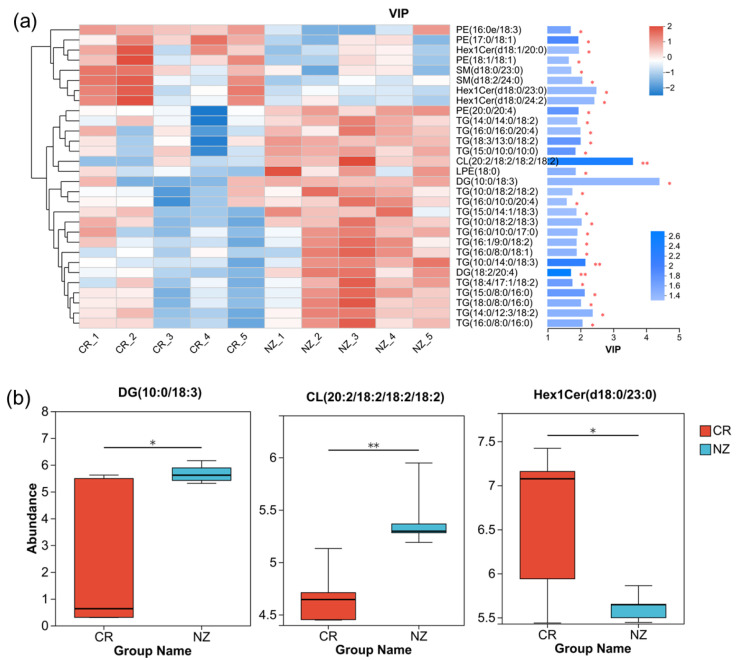
(**a**) VIP analysis plot of differential lipid metabolites with clustering and VIP values; (**b**) metabolite distribution box plot with the median of the relative abundance of the metabolite (central line). Significant differences are denoted as * *p* < 0.05 and ** *p* < 0.01.

To identify the potential biomarkers, the top 30 lipid metabolites with VIP values ≥1 were selected for clustering analysis, and the results are shown in Figure 5a. The top three abundant lipid metabolites were DG (10:0/18:3) (VIP = 4.39), CL (20:2/18:2/18:2/18:2) (VIP = 3.58), and Hex1Cer (d18:0/23:0) (VIP = 2.47), showing the most significant differences between CR and NZ. Figure 5b illustrates the relative abundance of the three metabolites in CR and NZ, respectively, with DG (10:0/18:3) and CL (20:2/18:2/18:2/18:2) being higher in the NZ group and Hex1Cer (d18:0/23:0) being higher in the CR group, albeit with greater data variability. The above-mentioned three lipid metabolites can serve as biomarkers to distinguish the rabbit meat from CR and NZ.

**Figure 6:**
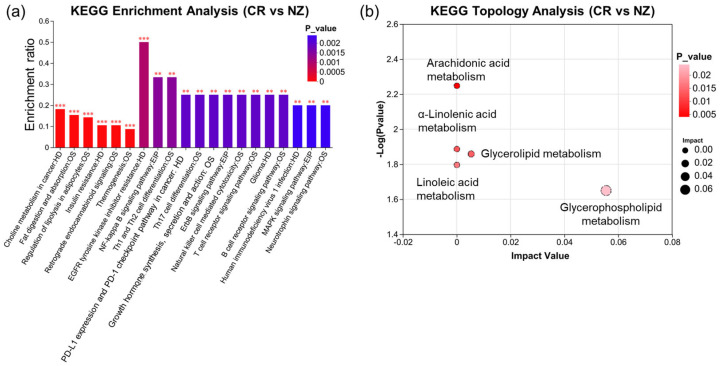
KEGG enrichment analysis of lipid metabolites in *longissimus dorsi* from Chuanbai Rex (CR) and New Zealand white (NZ) rabbits. (**a**) KEGG enrichment plot with pathway names (*X*-axis) and enrichment ratio (*Y*-axis); (**b**) KEGG topology analysis. Significant levels are denoted as ** *p* < 0.01 and *** *p* < 0.001.

KEGG pathway enrichment analysis was conducted to explore lipid pathway variations between the two breeds. Figure 6a shows that differential lipid metabolites between the CR and NZ groups were significantly enriched in 20 KEGG pathways. The “EGFR tyrosine kinase inhibitor resistance” pathway had the highest enrichment ratio (0.5, *p* < 0.001), while “Human immunodeficiency virus 1 infection,” “MAPK signaling pathway,” and “Linoleic acid metabolism” were also significantly enriched. Topological analysis using the Relative-Betweenness Centrality metric identified five major metabolic pathways (Figure 6b): glycerophospholipid metabolism, glycerolipid metabolism, linoleic acid metabolism, α-linolenic acid metabolism, and arachidonic acid metabolism. Notably, PC (15:0/18:2) participated in four pathways (glycerophospholipid, linoleic acid, α-linolenic acid, and arachidonic acid metabolism), and TG (16:0/16:0/20:4) was involved in glycerolipid metabolism. Glycerophospholipid metabolism was the most significant pathway, with an impact value of 0.0554.

Overall, lipidomic analysis of *longissimus dorsi* from two rabbit breeds indicated distinct patterns of lipid metabolites between CR and NZ. From 52 differential lipid metabolites, DG (10:0/18:3), CL (20:2/18:2/18:2/18:2), and Hex1Cer (d18:0/23:0) were identified as potential biomarkers for distinguishing the two breeds. The most enriched pathway was EGFR tyrosine kinase inhibitor resistance, and glycerophospholipid metabolism was the most significant metabolic pathway.

### 3.3. Analysis of Characteristic Volatile Organic Compounds (VOCs) in Rabbit Meat

**Figure 7:**
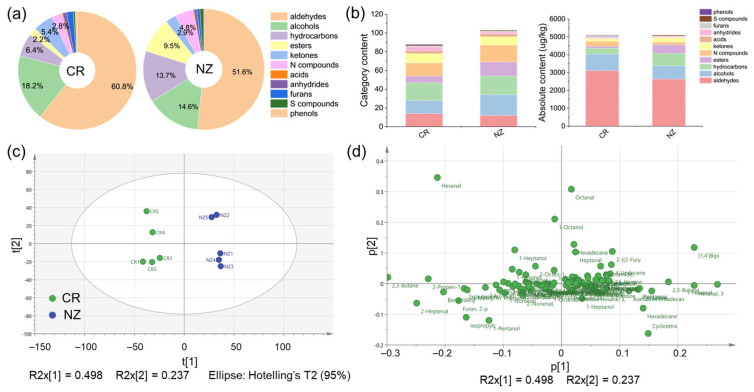
SPME-GC-MS analysis of characteristic volatile organic compounds (VOCs) in *longissimus dorsi* from Chuanbai Rex (CR) and New Zealand White (NZ) rabbits. (**a**) Pie chart of VOC percentages (%); (**b**) category content and absolute content (μg/kg) of VOCs; (**c**) PCA score plot; (**d**) PCA loading scatter plot.

SPME-GC-MS analysis detected a total of 140 volatile organic compounds (VOCs) in *longissimus dorsi* from CR and NZ, including 16 aldehydes, 26 alcohols, 27 hydrocarbons, 16 esters, 24 nitrogen-containing (N-) compounds, 18 ketones, 4 acids, 5 anhydrides, 2 furans, 1 sulfur-containing (S-) compound, and 1 phenol. The retention time, CAS number, and the absolute content of each VOC are summarized in Table S3. As shown in Figure 7a, the overall flavor profile of the two rabbit meats was predominantly composed of aldehydes, alcohols, and hydrocarbons, which made up 60.8%, 18.2%, and 6.4% in the CR, and 51.6%, 14.6%, and 13.7% in the NZ. Figure 7b indicates that the categories of VOCs were more abundant in the NZ than in the CR, but the total content of VOCs was comparable between the two breeds, which were 5110.8 μg/kg for CR and 5103.0 μg/kg for NZ, respectively. Comparably, the total content of aldehydes, alcohols, and ketones was higher in the CR (3106.4, 928.2, 276.8 μg/kg, respectively), while the content of hydrocarbons, esters, and N compounds was relatively higher in the NZ (698.1, 485.8, 245.7 μg/kg). The content of acids, anhydrides, furans, and S compounds was similar between the two breeds. As shown in Figure 7c, PCA showed that the two breeds are clearly separated, indicating significant differences in VOCs, with minor within-group variations. PC1 and PC2 accounted for 49.8% and 23.7% of the variance, respectively, with a cumulative contribution of 73.5%. The PCA loading plot (Figure 7d) highlighted the key VOCs that can be used to distinguish the VOCs between the two breeds, such as nonanal, heptanal, and octanal in the NZ (first and fourth quadrants). Furthermore, the OPLS-DA model (Figure S3) validated the PCA result and identified key VOCs, showing significant differences between CR and NZ. The Q^2^ and R^2^ values for the comparison were 0.54 and −0.774 (CR vs. NZ), respectively, indicating good model applicability and predictive capability. Permutation tests confirmed model reliability without overfitting, as indicated by negative Q2 intercepts.

**Figure 8:**
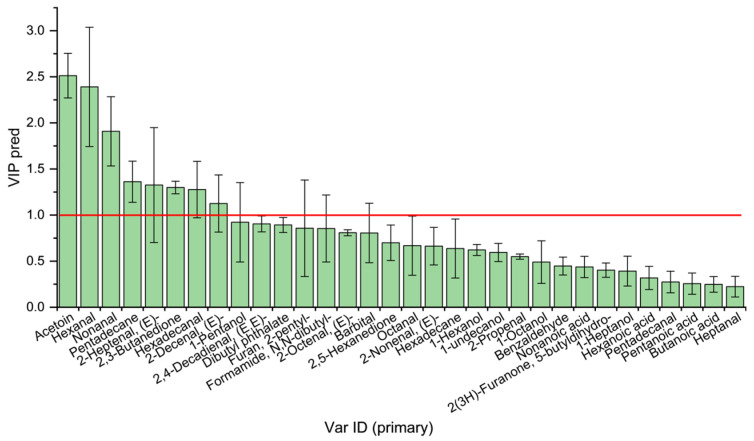
VIP values of key volatile organic compounds in rabbit meat from different breeds.

Multivariate analysis and odor activity values (OAVs) identified 31 key VOCs with OAV ≥ 1, and the odor description is summarized in Table S4. Of these, sixteen compounds were common to the two rabbit breeds, including eight aldehydes, four alcohols, one acid, two hydrocarbons, and one furan. VIP values were calculated to further identify key VOCs contributing significantly to flavor (Figure 8). Table 2 summarizes the eight key VOCs with VIP ≥ 1, whose category and content varied among the two breeds. Specifically, acetoin and 2-(E)-heptenal were unique to the CR, while 2,3-butanedione was unique to the NZ group. For the shared key VOCs, the NZ group had a higher content than the CR group. These results indicate significant differences in key VOCs among rabbit meat from different breeds.

### 3.4. Correlation of Lipid Metabolites with VOCs

**Figure 9:**
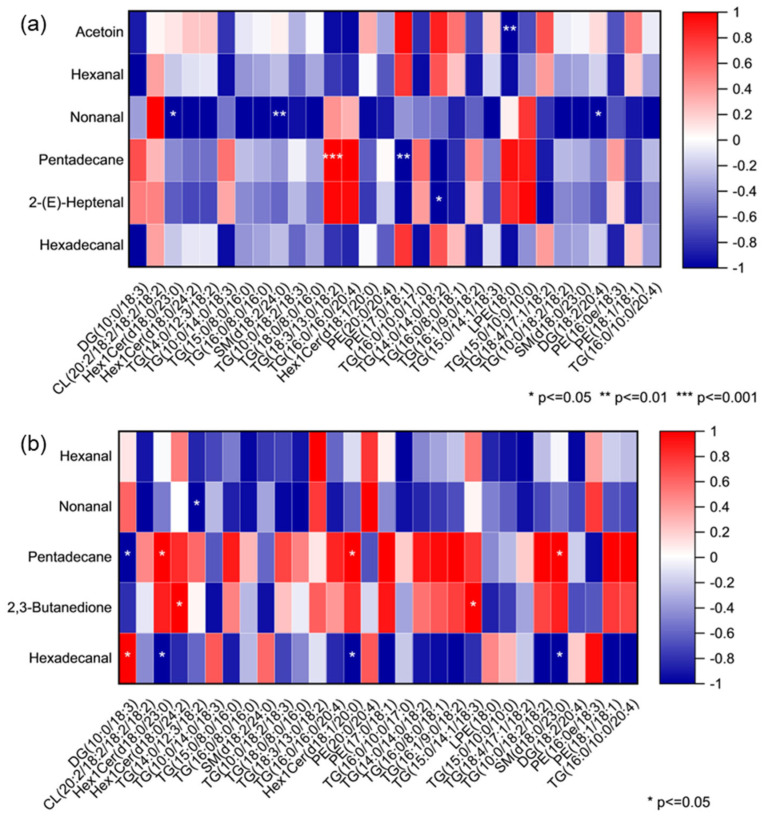
Correlation heatmap between the differential lipid metabolites and key volatile organic compounds in *longissimus dorsi* from (**a**) Chuanbai Rex and (**b**) New Zealand White rabbits.

To further elucidate the relationship between lipid composition and VOCs of rabbit meat, Pearson correlation analysis was conducted between differential lipid metabolites (VIP ≥ 1) and the characteristic VOCs (VIP ≥ 1) across the two breeds (Figure 9). The results revealed several notable associations, with select key correlations highlighted (* *p* < 0.05, ** *p* < 0.01, *** *p* < 0.001). As shown in Figure 9a, TG (18:3/13:0/18:2) showed a strong positive correlation with pentadecane in CR, which likely arises from the oxidative degradation of TG (18:3/13:0/18:2), or it served as a metabolic source for the formation of this long-chain alkane. In contrast, six other lipid metabolites were negatively correlated with four key VOCs in CR, including acetoin, nonanal, pentadecane, and 2-(E)-heptenal. This was probably because they are not direct precursors to the formation of these key VOCs. Instead, they may represent competing metabolic pathways or lipid pools that are less susceptible to the oxidation and degradation reactions that generate these specific aroma compounds.

From Figure 9b, in NZ, pentadecane was positively correlated with three lipids and negatively correlated with DG(10:0/18:3), whereas hexadecanal was negatively correlated with three lipids and positively correlated with DG(10:0/18:3). This correlation pattern shows significant difference to CR, likely due to the distinct lipid metabolic networks between two breeds, where DG(10:0/18:3) may serve as a key branching point, preferentially contributing to alkane formation in NZ while favoring aldehyde production in CR under different enzymatic or oxidative conditions.

## 4. Discussion

In recent years, China has promoted local rabbit breeds such as Chuanbai Rex (CR) for both fur and meat production [7]. CR was reported to have strong adaptability, fecundity, and disease resistance, and is increasingly used for producing processed products like roasted rabbit and jerky [3,8]. In comparison, the New Zealand white (NZ) rabbit is a well-established meat rabbit breed [5,33]. Thereby, this study aimed to identify the breed-specific differences in the lipids and VOCs of *longissimus dorsi* from the two breeds, thereby providing insights into improving the nutritional and sensory qualities of CR.

From fatty acid analysis, both CR and NZ are good sources of healthy PUFAs, with favorable PUFA/SFA ratios around 1.3–1.5, exceeding the United Kingdom Department of Health’s recommended ratio of ≥0.4 [34,35]. Linoleic acid (C18: 2n-6), palmitic acid (C16:0), oleic acid (C18: 1n9c), and stearic acid (C18:0) were found to be the most abundant species for CR and NZ, which agreed with previous studies [20,36]. Interestingly, CR was found to exhibit a more favorable n-6/n-3 PUFA ratio than NZ (5.07 versus 6.44), suggesting CR is richer in healthy PUFAs [37,38]. A previous study revealed that the fat from different parts of NZ (n = 12) had an n-6/n-3 PUFA ratio between 10.30 and 14.15 [36], which was not comparable to our result, probably because of the different diet fed to NZ. A similar comparative study was conducted by Zhang et al., and they found Fujian Yellow had a significantly lower n-6/n-3 PUFA ratio than NZ, though it contained more PUFAs (mainly arachidonic acid (C20:4n6)) and had a higher PUFA/SFA ratio [11].

Lipidomic profiling revealed 938 lipids in two breeds across five major classes: GL (485), GPs (303), SPs (145), FAs (3), and STs (2), and the most abundant lipid subclasses included TGs (347), PC (136), MePC (63), PE (60), which agreed with prior research [39,40]. A total of 52 differential lipid metabolites were identified between CR and NZ, with 19 upregulated (PE, PC) and 33 downregulated (mainly DG). Notably, PE and PC are relatively richer in CR, and they are primary substrates for lipid oxidation. It was reported that variations in PC and PE directly affected membrane fluidity and lipid oxidation pathways, thereby producing key aldehydes and ketones that determine the VOC profile and overall meat flavor [41]. Conversely, the downregulation of DG in CR is also functional because DG was proven to actively participate in GP synthesis [42,43]. The reduction in DG might indicate altered lipid metabolism and influence available precursors for flavor formation, potentially contributing to the distinct VOCs observed between the two breeds. The identified five major metabolic pathways also play a critical role in lipid homeostasis and facilitating membrane remodeling [44,45].

A total of thirty-one key VOCs with OAV ≥ 1 were identified in CR and NZ, including eight aldehydes, four alcohols, one acid, two hydrocarbons, and one furan, consistent with previous reports [27,46]. Among these, eight key VOCs with VIP ≥ 1 effectively distinguished the two breeds [47]. Notably, 2-(E)-heptenal (vanilla) and acetoin (buttery) were unique to the CR, whereas nonanal (wax/citrus/fresh/greasy/floral), pentadecane (waxy), and hexadecanal (cardboard) were more abundant in NZ. Correlation analysis revealed 20 lipids were associated with VOC formation, with distinct patterns between the two breeds. The PUFA-rich phospholipids serve as key precursors, undergoing hydrolysis and oxidative degradation to form aldehydes and ketones (e.g., C18:2 and C20:4 degraded into hexanal and nonanal, respectively) [15,48,49]. Previous metabolomic studies comparing Fujian Yellow and NZ rabbits identified 70 key KEGG pathways, with genes such as CPT2, PAK1, and FOXO6, correlated with metabolites including cholesterol and benzoic acid, further elucidating the metabolic bases of meat quality variation [11]. Future work should validate flavor differences through targeted measures of oxidative stability (e.g., TBARS, hexanal quantification) coupled with sensory evaluation, thereby clarifying the mechanistic links between lipid composition and VOC formation.

Recent studies have highlighted that the nutritional and sensory properties of rabbit meat are breed-specific. For example, CR rabbits exhibit a stronger antioxidant capacity than Hyla rabbits, contributing to a longer shelf life [50]. Comparative investigation of organoleptic properties has also revealed that NZ was superior than California White, but not comparable to Havana black rabbits in color, flavor, tenderness, and overall acceptability [51]. Furthermore, NZ was reported to contain higher fat content (2.81%) and better textural properties (chewiness and springiness) than Grey Flemish Giant rabbits [52]. Herein, we confirmed that CR possesses a favorable PUFA/SFA ratio and distinct VOCs compared to NZ, positioning it as a nutritious and potentially well-accepted meat source. The observed differences in lipids and VOCs were deduced to be largely determined by breed, but growth physiology and metabolic differences might also play a role due to the limited sample size.

We fully acknowledge that the sample size (n = 5 per breed) from one single farm is indeed small, which might limit the generalizability of statistical models and correlation pathways. The data obtained herein supported that the two breeds were biologically distinct, despite the small sample size. Overall, this work contributed to the establishment of a proof of concept that distinct lipidomic and volatilomic profiles exist between CR and NZ, and to pinpoint specific lipid-VOC relationships that serve as strong candidates for future validation. A follow-up study with a larger sample size, drawn from multiple farms and rearing systems, is essential to ensure robust biological replication. Future work can be performed by adding specific lipid precursors (e.g., linoleic or arachidonic acid) to the meat model and quantifying the resultant VOC formation to elucidate the reaction pathway of VOC formation. Integrated transcriptomics and sensory evaluation can be performed to further reveal the mechanisms underlying lipid–flavor relationships.

## 5. Conclusions

This study unraveled the relationship between key lipid metabolites and VOC formation in Chuanbai Rex (CR) and New Zealand white (NZ) rabbit meat (n = 5 per breed) through integrated lipidomic and volatilomic approaches, which could serve as a proof of concept for understanding the molecular mechanisms underlying flavor formation in rabbit meat. Results suggested that CR had a lower content of UFA/SFA but exhibited a more favorable PUFA/SFA ratio than NZ. A total of 52 differential lipid metabolites were identified between the two breeds, and DG (10:0/18:3), CL (20:2/18:2/18:2/18:2), and Hex1Cer (d18:0/23:0) were identified as potential biomarkers for distinguishment between the two breeds. SPME-GC-MS analysis revealed different VOCs, including acetoin and 2-(E)-heptenal that are unique in CR, while NZ was characteristic of nonanal and pentadecane. Correlation analysis indicated that six differential lipid metabolites were negatively correlated with four key VOCs (acetoin, nonanal, pentadecane, 2-(E)-heptanal) for CR, significantly different from the case of NZ. Overall, this preliminary comparative study could support the development of high-quality rabbit meat products tailored to consumer preferences. Future efforts can be devoted to elucidating the molecular and perceptual mechanisms underlying lipid–flavor interactions through integrated transcriptomics and sensory evaluation. Also, it is essential to further validate the identified correlation pathways through investigation on larger sample sizes across multiple farms, ages, and sexes.

## Figures and Tables

**Table 1:** Summary of medium- and long-chain fatty acids (MLCFAs) of *longissimus dorsi* from Chuanbai Rex (CR) and New Zealand white (NZ) rabbits.

Fatty Acids	CR (μg/mg)	NZ (μg/mg)	*p* Value	VIP
C8:0	0.0002 ± 0.0000	0.0002 ± 0.0001	>0.05	0.04
C10:0	0.0004 ± 0.0001 b	0.0005 ± 0.0000 a	0.031	0.06
C12:0	0.0012 ± 0.0004	0.0014 ± 0.0004	>0.05	0.10
C13:0	0.0010 ± 0.0001	0.0010 ± 0.0001	>0.05	0.03
C14:0	0.0298 ± 0.0100 b	0.0404 ± 0.0126 a	0.045	0.75
C15:0	0.0272 ± 0.0037	0.0271 ± 0.0023	>0.05	0.12
C15:1	0.0023 ± 0.0004	0.0022 ± 0.0004	>0.05	0.03
C16:0	0.3240 ± 0.0883 b	0.4280 ± 0.0944 a	0.03	2.47
C16:1	0.0066 ± 0.0036	0.0148 ± 0.0098	>0.05	0.69
C17:0	0.0139 ± 0.0037	0.0140 ± 0.0029	>0.05	0.17
C18:0	0.2220 ± 0.0459 b	0.3066 ± 0.0582 a	0.016	2.29
C18:1n9t	0.0283 ± 0.0019	0.0287 ± 0.0016	>0.05	0.16
C18:1n9c	0.3009 ± 0.0987 b	0.4062 ± 0.0907 a	0.015	2.30
C18:2n6t	0.0141 ± 0.0008	0.0141 ± 0.0004	>0.05	0.03
C18:2n6c	0.6470 ± 0.2250	0.7871 ± 0.1293	>0.05	2.10
C18:3n3	0.0880 ± 0.0494	0.0822 ± 0.0211	>0.05	0.19
C20:0	0.0018 ± 0.0005	0.0018 ± 0.0003	>0.05	0.01
C20:1n9	0.0246 ± 0.0032	0.0249 ± 0.0013	>0.05	0.10
C20:2	0.0184 ± 0.0005	0.0190 ± 0.0016	>0.05	0.21
C20:3n6	0.0228 ± 0.0008 b	0.0247 ± 0.0017 a	0.025	0.35
C20:4n6	0.0830 ± 0.0127 b	0.1149 ± 0.0213 a	0.047	1.59
C20:3n3	0.0226 ± 0.0011	0.0237 ± 0.0021	>0.05	0.27
C22:0	0.0014 ± 0.0002	0.0015 ± 0.0003	>0.05	0.02
C20:5n3	0.0227 ± 0.0004	0.0224 ± 0.0006	>0.05	0.01
C22:6n3	0.0179 ± 0.0005	0.0177 ± 0.0005	>0.05	0.05
∑	1.9218	2.4052	-	-
∑ SFA	0.6228	0.8226	-	-
∑ UFA	1.2990	1.5826	-	-
∑ MUFA	0.3626	0.4767	-	-
∑ PUFA	0.9363	1.1059	-	-
PUFA/SFA	1.5035	1.3444	-	-
n-6 PUFA	0.7668	0.9409	-	-
n-3 PUFA	0.1511	0.1460	-	-
n-6/n-3 ratio	5.07	6.44	-	-

Notes: (1) Raw data is summarized in Table S1; (2) significant differences were by ANOVA and Tukey’s HSD, which are represented by different lowercase letters with *p* < 0.05; (3) data presented are the mean ± standard deviation based on five biological replicates; (4) VIP values calculated according to PLSDA analysis between CR and NZ groups.

**Table 2:** Content of key volatile organic compounds in rabbit meat with VIP ≥ 1.

Compound	VIP	Content (μg/kg)	
		Chuanbai Rex	New Zealand White
Acetoin	2.51	270.3 ± 23.4	–
Hexanal	2.39	178.4 ± 5.4 b	194.0 ± 42.4 b
Nonanal	1.91	877.0 ± 202.8 b	1129.0 ± 265.3 a
Pentadecane	1.36	61.3 ± 22.2 b	121.7 ± 24.1 a
2-(E)-Heptenal	1.33	141.9 ± 49.6 a	–
2,3-Butanedione	1.30	–	86.7 ± 23.7
Hexadecanal	1.28	118.9 ± 23.9 b	173.5 ± 12.6 a
2-(E)-Decenal	1.13	–	–

Note: (1) Significant difference was represented by different lowercase letters with *p* < 0.05; (2) 95% CI for nonanal was (124.7, 1629.2) for CR and (390.6, 769.3) for NZ; 95% CI for pentadecane was (6.2, 116.4) for CR and (470.0, 1787.9) for NZ.

## Data Availability

The original contributions presented in this study are included in the article/Supplementary Materials. Further inquiries can be directed to the corresponding author.

## Supplementary Materials

The following supporting information can be downloaded at: https://www.mdpi.com/article/10.3390/foods14234024/s1, Figure S1: OPLSDA analysis of medium- and long-chain fatty acids of rabbit meat between Chuanbai Rex and New Zealand white; Figure S2: Lipid composition analysis of rabbit meat from Chuanbai Rex and New Zealand white rabbits. (a) distribution of carbon number of lipids; (b) distribution of double bond number of lipids; Figure S3: OPLS-DA of volatile organic compounds in the meat from Chuanbai Rex (CR) and New Zealand white (NZ) rabbits. (a) OPLS-DA score plot; (b) permutation validation model; Table S1: Raw data of medium- and long-chain fatty acids (MLCFA) of longissimus dorsi from Chuanbai Rex (CR) and New Zealand white (NZ) rabbits; Table S2: Summary of differential lipid metabolites between rabbit meat from Chuanbai Rex and New Zealand white; Table S3: Summary of volatile organic compounds (VOCs) in the meat from Chuanbai Rex (CR) and New Zealand white (NZ) rabbits; Table S4: Odor description and OAVs of key volatile organic compounds (VOCs) in the rabbit meat from Chuanbai Rex (CR) and New Zealand White (NZ).

## Abbreviations

The following abbreviations are used in this manuscript:

CR	Chuanbai Rex
NZ	New Zealand White
MLCFA	medium- and long-chain fatty acids
SFA	saturated fatty acids
UFA	unsaturated fatty acids
MUFA	monounsaturated fatty acids
PUFA	polyunsaturated fatty acids
PE	phosphatidylethanolamine
PC	phosphatidylcholine
SM	sphingomyelin
Hex1Cer	glycosylceramide
VOCs	volatile organic compounds
OAV	Ordor activity value
