# Supplementary material for: Unraveling the Relationship Between Lipids and Volatile Organic Compounds in Longissimus Dorsi of Chuanbai Rex and New Zealand White Rabbits
Source: Foods. 2025 Nov 24;14(23):4024. doi: 10.3390/foods14234024 (PMC12692007; doi:10.3390/foods14234024)
Supplement: Supplementary file 1 [file foods-14-04024-s001.zip › foods-3953823-supplementary.pdf]

Supporting Information

For

Unraveling the Relationship Between Lipids and Volatile Organic Compounds in *Longissimus Dorsi* of Chuanbai Rex and New Zealand White Rabbits

Meijun Zeng, Yang Li, Xiulian Wang, Ting Bai, Jie Cheng, Zhoulin Wu, Xiaohua Huang, Bo Wang, Rui Zhang, Jiamin Zhang * and Wei Wang

Meat Processing Key Laboratory of Sichuan Province, Sichuan Provincial Engineering Research Center of Meat Quality Improvement and Safety Control Technology, College of Food and Biological Engineering, Chengdu University, Chengdu 610106, China

* Correspondence: zhangjiamin@cdu.edu.cn

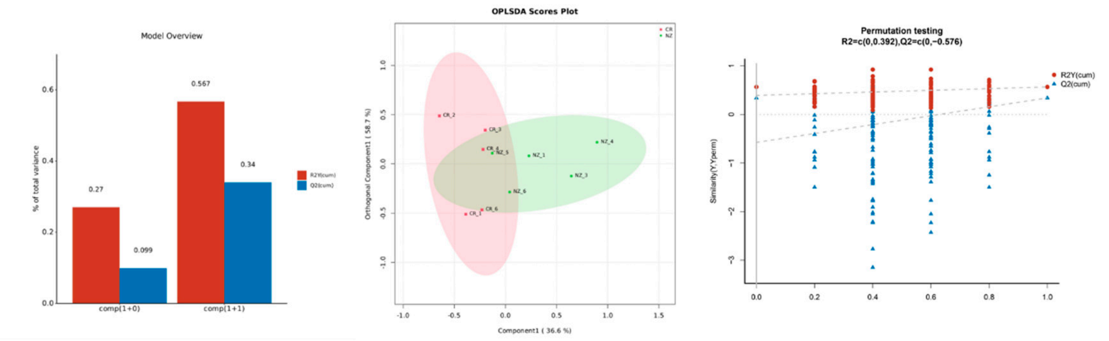

Figure S1. OPLSDA analysis of medium- and long-chain fatty acids of rabbit meat between Chuanbai Rex and New Zealand white.

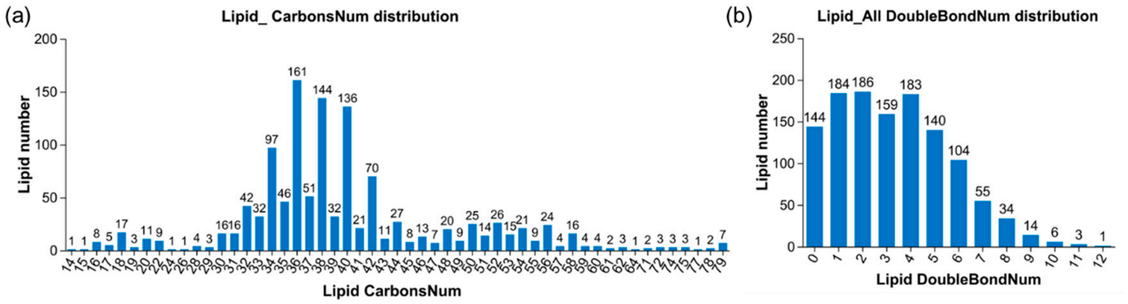

Figure S2. Lipid composition analysis of rabbit meat from Chuanbai Rex and New Zealand white rabbits. (a) distribution of carbon number of lipids; (b) distribution of double bond number of lipids.

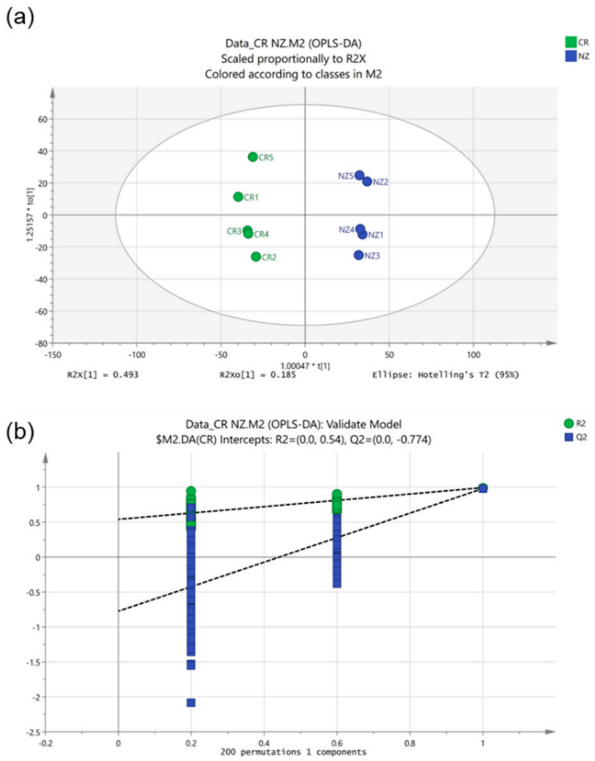

Figure S3. OPLS-DA of volatile organic compounds in the meat from Chuanbai Rex (CR) and New Zealand white (NZ) rabbits. (a) OPLS-DA score plot; (b) permutation validation model.

Table S1. Raw data of medium- and long-chain fatty acids (MLCFA) of *longissimus dorsi* from Chuanbai Rex (CR) and New Zealand white (NZ) rabbits

Metabolite	CR_1	CR_2	CR_3	CR_4	CR_5	NZ_1	NZ_2	NZ_3	NZ_4	NZ_5
C6:0	0.0000	0.0000	0.0000	0.0000	0.0000	0.0000	0.0000	0.0000	0.0000	0.0000
C8:0	0.0002	0.0002	0.0003	0.0002	0.0002	0.0002	0.0003	0.0003	0.0002	0.0002
C10:0	0.0004	0.0005	0.0005	0.0005	0.0003	0.0004	0.0006	0.0006	0.0005	0.0004
C11:0	0.0000	0.0000	0.0000	0.0000	0.0000	0.0000	0.0000	0.0000	0.0000	0.0000
C12:0	0.0008	0.0016	0.0013	0.0015	0.0007	0.0011	0.0015	0.0020	0.0013	0.0009
C13:0	0.0008	0.0012	0.0010	0.0011	0.0008	0.0010	0.0010	0.0012	0.0010	0.0009
C14:0	0.0195	0.0429	0.0311	0.0381	0.0174	0.0296	0.0500	0.0593	0.0375	0.0255
C14:1	0.0000	0.0000	0.0000	0.0000	0.0000	0.0000	0.0000	0.0000	0.0000	0.0000
C15:0	0.0225	0.0315	0.0285	0.0301	0.0232	0.0275	0.0274	0.0311	0.0255	0.0241
C15:1	0.0018	0.0027	0.0021	0.0028	0.0020	0.0023	0.0021	0.0028	0.0021	0.0016
C16:0	0.2134	0.4013	0.4012	0.3852	0.2187	0.4126	0.4672	0.5852	0.3664	0.3087
C16:1	0.0033	0.0099	0.0058	0.0115	0.0023	0.0050	0.0297	0.0216	0.0135	0.0043
C17:0	0.0086	0.0171	0.0168	0.0167	0.0103	0.0150	0.0131	0.0192	0.0124	0.0106
C17:1	0.0000	0.0000	0.0000	0.0000	0.0000	0.0000	0.0000	0.0000	0.0000	0.0000
C18:0	0.1587	0.2442	0.2839	0.2431	0.1799	0.3179	0.3077	0.4082	0.2510	0.2483
C18:1n9t	0.0259	0.0295	0.0299	0.0302	0.0260	0.0299	0.0289	0.0307	0.0279	0.0261
C18:1n9c	0.1695	0.3681	0.4054	0.3679	0.1935	0.3750	0.4295	0.5557	0.3956	0.2753
C18:2n6t	0.0132	0.0145	0.0152	0.0143	0.0133	0.0144	0.0137	0.0145	0.0136	0.0141
C18:2n6c	0.3308	0.8351	0.9063	0.7262	0.4365	0.8557	0.7621	0.9779	0.7550	0.5849
C18:3n6	0.0000	0.0000	0.0000	0.0000	0.0000	0.0000	0.0000	0.0000	0.0000	0.0000
C18:3n3	0.0327	0.1542	0.1189	0.1055	0.0287	0.0932	0.0656	0.1080	0.0940	0.0504
C20:0	0.0013	0.0021	0.0025	0.0018	0.0011	0.0018	0.0015	0.0022	0.0021	0.0014
C20:1n9	0.0205	0.0256	0.0238	0.0300	0.0232	0.0233	0.0246	0.0267	0.0237	0.0260
C20:2	0.0188	0.0182	0.0191	0.0182	0.0177	0.0186	0.0176	0.0220	0.0175	0.0193
C21:0	0.0000	0.0000	0.0000	0.0000	0.0000	0.0000	0.0000	0.0000	0.0000	0.0000
C20:3n6	0.0232	0.0218	0.0240	0.0227	0.0222	0.0260	0.0242	0.0269	0.0218	0.0249
C20:4n6	0.0669	0.0681	0.0941	0.0956	0.0902	0.1231	0.1307	0.1403	0.0839	0.0966
C20:3n3	0.0227	0.0219	0.0248	0.0219	0.0218	0.0235	0.0220	0.0274	0.0214	0.0241
C22:0	0.0014	0.0013	0.0017	0.0014	0.0013	0.0014	0.0012	0.0014	0.0021	0.0014
C20:5n3	0.0231	0.0219	0.0229	0.0226	0.0227	0.0226	0.0216	0.0229	0.0219	0.0230
C22:1n9	0.0000	0.0000	0.0000	0.0000	0.0000	0.0000	0.0000	0.0000	0.0000	0.0000
C22:2n6	0.0000	0.0000	0.0000	0.0000	0.0000	0.0000	0.0000	0.0000	0.0000	0.0000
C23:0	0.0000	0.0000	0.0000	0.0000	0.0000	0.0000	0.0000	0.0000	0.0000	0.0000
C24:0	0.0000	0.0000	0.0000	0.0000	0.0000	0.0000	0.0000	0.0000	0.0000	0.0000
C24:1n9	0.0000	0.0000	0.0000	0.0000	0.0000	0.0000	0.0000	0.0000	0.0000	0.0000
C22:6n3	0.0182	0.0172	0.0186	0.0180	0.0174	0.0180	0.0177	0.0179	0.0167	0.0182

Table S2. Summary of differential lipid metabolites between rabbit meat from Chuanbai Rex and New Zealand white

Lipid	Regulate	VIP$^{OPLS-DA}$	VIP^{PLS-DA}	Category	Class	Class name
LPE(18:0)	down	1.8444	1.5225	GP	LPE	lysophosphatidylethanolamine
PE(18:0/20:4)	down	1.4217	1.257	GP	PE	phosphatidylethanolamine
PC(15:0/18:2)	up	1.0851	0.8627	GP	PC	phosphatidylcholine
PE(18:1/18:1)	up	1.6339	1.3819	GP	PE	phosphatidylethanolamine
PE(18:3e/20:4)	up	1.2924	1.066	GP	PE	phosphatidylethanolamine
PC(17:0/18:1)	up	1.4399	1.2783	GP	PC	phosphatidylcholine
PE(17:0/18:1)	up	1.9241	1.5506	GP	PE	phosphatidylethanolamine
PE(18:0/20:3)	down	1.1301	0.9675	GP	PE	phosphatidylethanolamine
PC(16:0e/20:3)	down	1.4451	1.2502	GP	PC	phosphatidylcholine
PE(20:0/20:4)	down	1.9317	1.5975	GP	PE	phosphatidylethanolamine
dMePE(16:0/20:4)	down	1.4122	1.2365	GP	dMePE	dimethylphosphatidylethanolamine
PE(16:0e/18:3)	up	1.6931	1.4681	GP	PE	phosphatidylethanolamine
dMePE(15:0/18:2)	up	1.2149	1.0185	GP	dMePE	dimethylphosphatidylethanolamine
PE(18:1/22:2)	down	1.5625	1.2451	GP	PE	phosphatidylethanolamine
CL(20:2/18:2/18:2/18:2)	down	3.5839	2.925	GP	CL	Cardiolipin
Cer(t16:0/26:0)	up	1.1263	0.9143	SP	Cer	Ceramides
DG(18:1/18:2)	down	1.0105	0.8651	GL	DG	diglyceride
PC(17:0/18:2)	up	1.1645	0.9662	GP	PC	phosphatidylcholine
PC(16:1/19:0)	up	1.1291	0.958	GP	PC	phosphatidylcholine
TG(14:0/18:2/18:3)	down	1.4889	1.2574	GL	TG	triglyceride
TG(14:0/14:0/18:2)	down	1.893	1.5602	GL	TG	triglyceride
SM(d19:1/18:1)	up	1.4118	1.2094	SP	SM	sphingomyelin
SM(d18:1/24:3)	up	1.0465	0.8762	SP	SM	sphingomyelin
TG(18:3/13:0/18:2)	down	1.9935	1.5744	GL	TG	triglyceride
TG(16:0/8:0/18:1)	down	1.874	1.6625	GL	TG	triglyceride
TG(16:0/8:0/16:0)	down	2.0521	1.6515	GL	TG	triglyceride
TG(16:0/16:1/18:3)	down	1.445	1.2445	GL	TG	triglyceride
TG(10:0/18:2/18:2)	down	1.7435	1.5156	GL	TG	triglyceride
SM(d19:0/18:1)	up	1.4219	1.1458	SP	SM	sphingomyelin
TG(10:0/18:2/18:3)	down	2.0258	1.6334	GL	TG	triglyceride
Hex1Cer(d18:1/20:0)	up	1.9402	1.7077	SP	Hex1Cer	Simple Glc series

DG(22:4/18:2)	down	1.3466	1.0368	GL	DG	diglyceride
DG(20:2/18:2)	down	1.228	0.9299	GL	DG	diglyceride
TG(15:0/8:0/16:0)	down	2.12	1.7666	GL	TG	triglyceride
PC(18:1/14:0)	up	1.2277	1.0127	GP	PC	phosphatidylcholine
TG(15:0/14:1/18:3)	down	1.8683	1.5433	GL	TG	triglyceride
DG(20:3/18:2)	down	1.2429	1.0367	GL	DG	diglyceride
DG(18:2/20:4)	down	1.7016	1.376	GL	DG	diglyceride
TG(16:0/16:0/20:4)	down	1.9908	1.5345	GL	TG	triglyceride
TG(16:1/9:0/18:2)	down	1.8714	1.5221	GL	TG	triglyceride
SM(d18:0/23:0)	up	1.7122	1.7938	SP	SM	sphingomyelin
TG(16:0/10:0/20:4)	down	1.5727	1.3746	GL	TG	triglyceride
SM(d18:2/24:0)	up	2.0444	2.2276	SP	SM	sphingomyelin
TG(15:0/10:0/10:0)	down	1.8376	1.4091	GL	TG	triglyceride
DG(10:0/18:3)	down	4.3925	3.5883	GL	DG	diglyceride
Hex1Cer(d18:0/23:0)	up	2.4741	2.4851	SP	Hex1Cer	Simple Glc series
Hex1Cer(d18:0/24:2)	up	2.4115	2.4682	SP	Hex1Cer	Simple Glc series
TG(18:0/8:0/16:0)	down	2.0051	1.6546	GL	TG	triglyceride
TG(10:0/14:0/18:3)	down	2.1395	1.7625	GL	TG	triglyceride
TG(16:0/10:0/17:0)	down	1.897	1.5218	GL	TG	triglyceride
TG(14:0/12:3/18:2)	down	2.3595	1.9118	GL	TG	triglyceride
TG(18:4/17:1/18:2)	down	1.7462	1.4803	GL	TG	triglyceride

Notes: the identified differential lipids were all significantly different with VIP_pred_OPLS-DA > 1, $p < 0.05$, fold change (FC) >1.

Table S3. Summary of volatile organic compounds (VOCs) in the meat from Chuanbai Rex (CR) and New Zealand white (NZ) rabbits.

Category	No.	RT	Compound	CAS no.	CR	NZ
aldehydes	1	4.33	Hexanal	66-25-1	178.4 ± 5.4	194.0 ± 42.4
	2	5.32	Hexanal, 3-methyl-	19269-28-4	71.7 ± 10.6	154.3 ± 16.8
	3	5.35	Heptanal	111-71-7	180.2 ± 5.9	194.9 ± 42.4
	4	6.53	Octanal	124-13-0	361.4 ± 113.7	382.9 ± 80.8
	5	7.81	Nonanal	124-19-6	877.0 ± 302.0	1129.0 ± 265.3pen
	6	7.05	2-Heptenal, (E)-	18829-55-5	141.9 ± 49.6	–
	7	7.20	2-Propenal	107-02-8	–	15.8 ± 3.5
	8	9.76	Benzaldehyde	100-52-7	230.5 ± 64.6	167.7 ± 15.9
	9	9.88	2-Nonenal, (E)-	18829-56-6	–	58.2 ± 8.9
	10	13.67	Benzaldehyde, 3-ethyl-	34246-54-3	40.0 ± 9.7	13.1 ± 4.6
	11	14.58	2-Octenal, (E)-	2548-87-0	85.9 ± 14.2	58.2 ± 5.9
	12	14.58	2-Undecenal	2463-77-6	69.0 ± 12.3	–
	13	15.78	2,4-Decadienal, (E,Z)-	25152-83-4	40.9 ± 6.8	–
	14	18.94	Benzaldehyde, 3-methyl-	620-23-5	230.5 ± 98.5	–
	15	19.16	Pentadecanal	2765-11-9	17.9 ± 2.4	17.8 ± 6.1
	16	20.47	Hexadecanal	629-80-1	118.9 ± 23.9	173.5 ± 12.6
				total	3106.4	2635.4
alcohols	1	5.43	1-Butanol, 3-methyl-	123-51-3	102.0 ± 15.8	–
	2	5.96	1-Pentanol	71-41-0	36.1 ± 5.1	31.1 ± 4.6
	3	6.84	(2-Methyloxan-2-yl)methanol	1000436-45-7	–	17.3 ± 5.8
	4	7.21	1-Hexanol	111-27-3	73.1 ± 12.4	21.1 ± 6.0
	5	8.50	1-Heptanol	111-70-6	122.3 ± 29.9	101.1 ± 27.9
	6	8.53	1-Pentanol, 4-methyl-	626-89-1	–	24.6 ± 7.5
	7	9.02	1-Hexanol, 2-ethyl-	104-76-7	–	138.7 ± 36.4
	8	9.23	Cyclohexaneethanol	4442-79-9	–	22.1 ± 6.1
	9	9.24	Cyclohexanemethanol	100-49-2	16.6 ± 5.2	–
	10	9.77	2,3-Butanediol,[S-(R,R)]	19132-06-0	219.6 ± 64.3	–
	11	10.07	1-Octanol	111-87-5	261.5 ± 21.5	262.5 ± 74.9
	12	11.18	Cyclooctyl alcohol	696-71-9	–	13.1 ± 5.3
	13	11.19	2-Octen-1-ol, (Z)-	26001-58-1	33.5 ± 8.4	28.3 ± 11.0
	14	12.18	1-Nonanol	143-08-8	23.8 ± 9.8	10.4 ± 2.9
	15	12.18	1-Heptanol, 4-methyl-	817-91-4	9.2 ± 2.4	–
	16	12.19	6-Methyl-1-octanol	110453-78-6	17.7 ± 5.1	8.4 ± 3.2
	17	14.58	1-Undecanol	112-42-5	–	26.4 ± 6.0
	18	17.55	Benzenemethanol	98-85-1	4.5 ± 0.8	–

	19	18.30	1-Pentanol, 3-methyl-	589-35-5	–	4.6 ± 2.1
	20	18.30	1-Dodecyn-4-ol	74646-36-9	–	1.6 ± 0.5
	21	18.31	(S)-(+)-5-Methyl-1-heptanol	57803-73-3	7.7 ± 1.2	4.3 ± 1.1
	22	19.16	trans-2-Dodecen-1-ol	69064-37-5	–	17.0 ± 4.5
	23	20.86	3,4-Dimethyl-5-hexen-3-ol	1569-45-5	–	1.8 ± 0.6
	24	22.98	1,14-Tetradecanediol	19812-64-7	–	8.4 ± 3.1
	25	22.98	trans-3-Methylcyclohexanol	7443-55-2	–	10.0 ± 4.8
	26	23.35	1-Hexanol, 4-methyl	818-49-5	0.6 ± 0.3	–
				total	928.2	742.6
hydrocarbons	1	3.76	Neopentane	463-82-1	–	5.8 ± 2.6
	2	4.32	Pentane, 3-methyl-	96-14-0	–	323.7 ± 55.8
	3	7.06	4-Undecene, 3-methyl-, (Z)-	74645-87-7	–	25.8 ± 6.4
	4	7.21	Oxirane, 2,3-bis(1-methylethyl)-, trans-	54644-32-5	38.1 ± 10.3	–
	5	8.50	Isopropylcyclobutane	872-56-0	93.3 ± 25.8	–
	6	8.98	1-Pentene, 4,4-dimethyl-	762-62-9	31.8 ± 8.4	–
	7	9.07	Pentadecane	629-62-9	61.3 ± 22.2	121.7 ± 24.1
	8	9.23	1-Tridecyne	26186-02-7	29.2 ± 7.1	–
	10	9.78	Methylal	109-87-5	76.3 ± 25.8	–
	11	9.88	4-Decene, 3-methyl-, (E)-	62338-47-0	–	8.1 ± 2.6
	12	10.75	Hexadecane	544-76-3	17.7 ± 6.3	57.2 ± 32.8
	13	10.76	Hexane, 3,3-dimethyl-	563-16-6	15.3 ± 5.0	32.5 ± 7.6
	14	10.76	Undecane, 3,8-dimethyl-	17301-30-3	–	36.5 ± 9.1
	15	12.19	1-Heptene, 6-methyl-	5026-76-6	–	8.1 ± 2.9
	16	13.07	Butane, 2,2-dimethyl-	75-83-2	–	8.1 ± 3.7
	17	13.07	Heptadecane	629-78-7	–	0.7 ± 0.3
	18	14.58	Cyclopentane, 1-ethyl-1-methyl-	16747-50-5	11.6 ± 0.3	6.6 ± 2.3
	19	14.58	1-Decene, 8-methyl-	61142-79-8	1.9 ± 0.6	4.7 ± 1.8
	20	14.58	1-Undecene, 9-methyl-	74630-41-4	–	12.9 ± 3.6
	21	18.31	1-Octene, 6-methyl-	13151-10-5	6.3 ± 2.0	5.8 ± 1.5
	22	18.37	Naphthalene, 2,7-dimethyl-	582-16-1	–	4.8 ± 0.8
	24	21.67	Cyclohexane, undecyl-	54105-66-7	4.6 ± 1.3	–
	25	23.35	Cyclopentane, 1-methyl-3-(2-methylpropyl)-	29053-04-1	–	10.0 ± 0.6
	26	23.36	3-Hexene, 2,2-dimethyl-	3123-93-1	–	2.3 ± 0.8
	27	23.37	4-Nonene, 3-methyl-, (Z)-	63830-69-3	–	4.2 ± 1.7
				total	326.4	698.1
esters	1	5.96	Oxetane, 3-(1-methylethyl)-	10317-17-6	–	15.5 ± 4.6
	2	6.83	n-Caproic acid vinyl ester	3050-69-9	26.7 ± 12.3	8.2 ± 3.2

	3	7.05	Oxalic acid, cyclohexyl isobutyl ester	1000309-30-4	–	8.5 ± 2.9
	4	7.06	Oxalic acid, cyclohexyl butyl ester	1000309-30-5	–	36.3 ± 7.9
	5	7.23	2-Oxetanone, 4-methyl-	3068-88-0	–	5.6 ± 1.6
	6	9.23	4-Hexen-1-ol, acetate, (Z)-	42125-17-7	–	18.3 ± 6.5
	7	10.74	2-Propenoic acid, 2-methyl-, 2-methylpropyl ester		6.0 ± 1.2	–
	8	11.19	3-Hexen-1-ol, propanoate, (Z)-	33467-74-2	10.7 ± 0.2	–
	10	12.19	2-Methylpentyl formate	381670-34-4	–	5.3 ± 2.1
	11	19.27	5-Oxotetrahydrofuran-2-carboxylic acid, ethyl ester	1126-51-8	7.6 ± 2.1	–
	12	19.27	2(3H)-Furanone, dihydro-5-propyl-	105-21-5	10.0 ± 3.7	5.4 ± 1.8
	13	19.28	2(3H)-Furanone, 5-butyldihydro-	104-50-7	8.1 ± 2.7	–
	14	21.81	Dibutyl phthalate	84-74-2	40.1 ± 17.9	–
	15	21.81	1,4-Dibutyl benzene-1,4-dicarboxylate	1962-75-0	–	31.9 ± 12.8
	16	21.81	Phthalic acid, butyl hexyl ester	1010308-99-5	–	24.0 ± 9.7
				total	110.0	485.8
ketones	1	5.32	2,3-Butanedione	431-03-8	–	86.7 ± 23.7
	2	6.52	Acetoin	513-86-0	270.3 ± 23.4	–
	3	6.84	2,5-Hexanedione	110-13-4	–	23.8 ± 5.2
	4	6.88	2,5-Hexanedione	110-13-4	–	11.7 ± 5.4
	5	7.02	2H-Pyran-2-one, 5,6-dihydro-6-propyl-	16400-69-4	–	11.4 ± 2.9
	6	7.05	1,5-Heptadien-4-one, 3,3,6-trimethyl-	546-49-6	4.8 ± 1.8	–
	7	11.18	1,3-Benzodioxol-2-one, hexahydro-, trans-	20192-66-9	10.4 ± 3.1	–
	8	12.08	2-Methylhept-6-en-3-one	1000424-30-2	11.5 ± 5.2	–
	10	13.54	1,3-Benzodioxol-2-one, hexahydro-, trans-	20192-66-9	–	3.6 ± 0.9
	11	14.81	Paramethadione	115-67-3	–	1.2 ± 0.4
	12	15.89	3(2H)-Furanone, 2-hexyl-5-methyl-	33922-66-6	1.4 ± 0.6	–

	13	15.84	3(2H)-Furanone, 5-methyl-2-octyl-	57877-72-2	–	3.1 ± 0.9
	14	16.47	Bicyclo[3.1.0]hex-3-en-2-one, 5-(1-methylethyl)-	36262-12-1	–	1.6 ± 0.5
	15	16.94	4,5-Octanedione	5455-24-3	–	1.6 ± 0.7
	16	19.63	2(3H)-Furanone, 5-acetyldihydro-	29393-32-6	5.1 ± 2.1	–
	17	20.26	2,5-Dihydroxy-4-methoxyacetophenone	1000422-88-0	1.3 ± 0.4	–
	18	20.31	3(2H)-Furanone, 4-hydroxy-5-methyl-	19322-27-1	1.3 ± 0.3	–
				total	276.8	145.7
N compounds	1	3.11	4-Methyl-1,3-oxazolidin-2-one	4042-43-7	6.7 ± 2.7	–
	2	3.17	N,N-Diethylheptylamine	1000391-35-1	16.5 ± 5.3	–
	3	3.29	3-Methyl-2-phenyl-1H-pyrrolo[2,3-b]pyridine	139962-70-2	1.9 ± 0.6	–
	4	3.75	n-Hexylmethylamine	35161-70-7	–	10.7 ± 3.7
	5	5.86	Furfurylmethylamphetamine	13445-60-8	29.7 ± 5.3	25.7 ± 6.8
	6	5.90	1,2,4,5-Tetrazine, 3,6-dipropyl-	13717-92-5	–	12.5 ± 2.0
	7	6.03	2-Propen-1-amine	107-11-9	11.8 ± 4.2	–
	8	7.64	Pyridazine	289-80-5	–	58.0 ± 18.5
	9	8.99	Azetidine	503-29-7	–	28.3 ± 8.2
	10	9.87	Cyclohexane, nitro-	1122-60-7	–	19.3 ± 3.1
	11	12.41	3,5-Diamino-1,2,4-triazole	1455-77-2	–	0.9 ± 0.1
	12	13.57	Glycyl-dl-alanine	926-77-2	7.3 ± 5.6	4.7 ± 1.5
	13	14.43	Oxime-, methoxy-phenyl-_	1000222-86-6	–	12.5 ± 4.9
	14	15.15	Formamide, N,N-dibutyl-	761-65-9	34.5 ± 14.1	27.5 ± 9.6
	15	15.15	2,4-Dioxohexahydro-1,3,5-triazine	1000484-54-9	13.2 ± 6.4	–
	16	15.84	3-Methyl-1-[(1H)-1,2,4-triazol-1-yl]butan-2-one	64922-02-7	–	3.6 ± 1.0
	17	16.68	1-Methyl-1H-1,2,4-triazole	6086-21-1	1.3 ± 0.3	–
	18	17.64	Hexanenitrile, 5-methyl-	19424-34-1	–	6.3 ± 2.6
	19	18.37	Barbital	57-44-3	–	3.1 ± 1.1
	20	18.92	Probarbital	143-82-8	–	3.5 ± 1.3
	21	18.94	2,4-imidazolidinedione, 3-phenyl-	1000404-72-6	3.5 ± 2.0	1.0 ± 0.6

	22	20.34	2,4,6,(1H,3H,5H)-Pyrimidinetrione, 5-acetyl-	58713-02-3	–	2.8 ± 1.3
	23	24.93	4-Pyridinecarboxamide	1453-82-3	–	10.0 ± 2.6
	24	24.93	Niacinamide	98-92-0	16.7 ± 7.7	15.2 ± 2.9
				total	143.3	245.7
acids	1	16.40	Pentanoic acid	109-52-4	3.2 ± 0.7	3.5 ± 0.7
	2	16.39	Butanoic acid	107-92-6	3.9 ± 0.8	4.5 ± 1.9
	3	16.40	Hexanoic acid	142-62-1	7.8 ± 1.8	–
	4	20.84	Nonanoic acid	112-05-0	–	6.0 ± 1.6
				total	15.0	14.0
anhydrides	1	6.89	2-Methylpentanoic anhydride	63169-61-9	4.4 ± 0.9	1.8 ± 0.8
	2	11.83	Hexanoic acid, anhydride		4.2 ± 0.8	–
	3	12.20	Valeric anhydride	2082-59-9	–	2.0 ± 0.3
	4	17.66	2-Methylbutanoic anhydride	1468-39-9	6.0 ± 1.6	–
	5	18.37	Barbital	57-44-3	41.2 ± 6.0	–
				total	55.8	3.8
furans	1	5.78	Furan, 2-pentyl-	3777-69-3	93.2 ± 25.9	–
	2	5.79	2-[(2-Furylmethyl)amino]-2-methyl-1-propanol	889949-94-4	–	39.6 ± 24.9
phenols	1	16.48	Phenol, 4-(1-methylpropyl)-	99-71-8	30.9 ± 12.1	–
S compounds	1	17.33	Dimethyl sulfone	67-71-0	16.1 ± 3.3	22.5 ± 5.4

Notes: RT denoted retention time in SPME-GC-MS analysis. Data presented were mean ± standard deviation based on five biological replicates.

Table S4. Odor description and OAV values of key volatile organic compounds (VOCs) in the rabbit meat from Chuanbai Rex (CR) and New Zealand White (NZ).

No.	Compound	CAS#	threshold (ug/kg)	odor description	OAV	
					CR	NZ
1	Hexanal	66-25-1	0.005	fresh grass/vanilla/fruit	229522.7	115995.0
2	Heptanal	111-71-7	0.0028	nutty/fruit	63698.3	69295.0
3	Octanal	124-13-0	0.00059	fresh/greasy/fruit/honey	707121.2	670889.4
4	Nonanal	124-19-6	0.0011	wax/citrus/fresh/greasy/floral	797233.3	1026353.3
5	2-Octenal, (E)-	2548-87-0	0.003	greasy	2476.1	–
6	2-Nonenal, (E)-	18829-56-6	0.00019	greasy/herb	348961.2	254322.7
7	2-Heptenal, (E)-	18829-55-5	0.04	vanilla	3546.3	–
8	2-Propenal	107-02-8	0.23	fruity/almond/cherry	–	63.0
9	Benzaldehyde	100-52-7	0.75089	almond/nutty	293.7	230.3
10	2,4-Decadienal, (E,E)-	25152-84-5	0.0003	orange	120458.1	–
11	Pentadecanal	2765-11-9	1	sweet	17.9	17.0
12	Hexadecanal	629-80-1	60	cardboard	2.0	2.9
13	1-Pentanol	71-41-0	10	sweet	2.9	2.5
14	1-Hexanol	111-27-3	0.0056	fresh/fruit/sweet	13045.3	3768.1
15	1-Heptanol	111-70-6	0.24	pungent	458.5	475.4
16	1-Octanol	111-87-5	0.1258	greasy/orange/rose	2050.9	2035.6
17	1-undecanol	112-42-5	5	citrus	–	4.8
18	Vinyl hexanoate	3050-69-9	0.003	fruity/pineapple	8893.4	–
19	2(3H)-Furanone, 5-butyldihydro-	104-50-7	6.5	sweet/fruity/coconut	1.8	–
20	Dibutyl phthalate	84-74-2	2.6	aromatic	14.3	–
21	Acetoin	513-86-0	14	buttery	19.3	–
22	2,3-Butanedione	431-03-8	30	buttery/fruity	–	2.9
23	2,5-Hexanedione	110-13-4	5	sweet aromatic	–	3.9
24	Butanoic acid	107-92-6	2.4	cream	1.3	1.9
26	Hexanoic acid	142-62-1	0.89	cheese	9.3	–
28	Pentadecane	629-62-9	60	waxy	1.0	2.0
29	Hexadecane	544-76-3	60	mild waxy	0.3	1.0
30	Furan, 2-pentyl-	3777-69-3	5.8	rice	19.1	–
31	Barbital	57-44-3	40	bitter	1.1	0.1
